# Resistive states in strontium titanate thin films: Bias effects and mechanisms at high and low temperature

**DOI:** 10.1007/s10832-017-0081-2

**Published:** 2017-04-03

**Authors:** M. Kubicek, S. Taibl, E. Navickas, H. Hutter, G. Fafilek, J. Fleig

**Affiliations:** Institute of Chemical Technologies and Analytics, TU Wien, Getreidemarkt, 9-164/EC, 1060 Vienna, Austria

**Keywords:** Strontium titanate, STO, Defect chemistry, Resistive switching, Mixed ionic electronic conduction

## Abstract

A study on charge transport properties of thin film Fe-doped SrTiO_3_ epitaxially grown on Nb-doped SrTiO_3_ is reported. Electric measurements between 350 °C and 750 °C show a transition from predominant ionic to electronic conduction and lower conductivity of the thin films compared to the bulk of polycrystalline samples. Defect chemical changes at elevated temperature were investigated by applying a bias voltage. A model is described which successfully predicts additional features such as inductive loops or extra semicircles measureable by impedance spectroscopy as well as the complicated time dependence of electric DC-measurements. With this model it is also possible to calculate the negligibly small ionic conductivity next to the dominating electronic conductivity in the high temperature regime. The ionic conductivity is referenced by oxygen isotope depth profiling. Changes of resistive states in Fe-doped SrTiO_3_ thin films at high temperature and moderate fields are compared to room temperature resistive switching phenomena at high electric fields. A conductive filament based switching process is observed at room temperature, and the capability for forming such filaments and their electric properties is further analysed using microelectrodes.

## Introduction

Strontium titanate is a model electroceramic and among the best investigated oxide materials [1, 2]. It adopts the cubic perovskite structure ABO_3_ and crystallizes in space group $Pm\overline{3} m$. Nominally undoped SrTiO_3_ behaves like a slightly p-doped material, but by varying temperature and oxygen partial pressure, its electric conductivity can be changed between predominantly electronic via either electrons or electron holes or predominantly ionic via oxygen vacancies [3–7]. The oxygen exchange reaction at the surface and a resulting change in oxygen non-stoichiometry *δ* in SrTiO_3-*δ*_ governs these transitions at intermediate temperatures [4]. At high temperatures also variations in cation vacancy concentrations can play a role [5]. The defect concentrations can be further modified by doping SrTiO_3_ with either acceptors or donors (e.g. Fe^3+^ or Nb^5+^ on the Ti^4+^ site). This is regularly done to achieve certain well defined electrical properties [6]. Mixed ionic electronic conductivity makes especially p-doped SrTiO_3_ an interesting material for solid oxide fuel cell electrodes and for sensors [8–12].

Besides these bulk conductivity mechanisms, SrTiO_3_ is also known for interesting electric properties at interfaces. The most well-known example is the highly conductive 2-dimensional electron gas at the SrTiO_3_|LaAlO_3_ interface [13, 14]. Strontium titanate is also a good dielectric with a high relative permittivity of about ε = 300 at room temperature. In contrast to closely related BaTiO_3_, which is ferroelectric below 120 °C, SrTiO_3_ exhibits a cubic unit cell between room temperature and its melting point. However, the transition to ferroelectric SrTiO_3_ can be induced by external forces such as lattice strain [15]. SrTiO_3_ has a bandgap of about 3.3 eV at room temperature [16]. Therefore, ultraviolet light can excite valence band electrons to the conduction band and induces photoconductivity [17, 18] or substantial photovoltages at interfaces, even above 400 °C [19]. Moreover, an enhancing effect of UV-light on the oxygen exchange kinetics was found [20]. Besides TiO_2_, SrTiO_3_ is also among the most important materials for photocatalysis [21, 22]. Furthermore, SrTiO_3_ is a well investigated active material for non-volatile resistive switches [23–27]. Here, two or more non-volatile resistive states can be addressed by large voltage or current pulses and read out at low currents without changing the resistive state.

We can summarize that SrTiO_3_ is an electroceramic which can be altered in its properties and resistive states by many factors. However, despite all these studies neither interfacial effects nor properties of SrTiO_3_ thin films are completely understood. Thin films exhibit conductivities that differ from bulk SrTiO_3_ [28, 29]. These differences can occur due to several reasons such as space charges at grain boundaries or dislocations or cationic defects introduced at growth which might also affect switching phenomena upon bias voltage [24, 30–32]. In this paper we investigate 0.37 mol% Fe-doped SrTiO_3_ epitaxial thin films grown on 0.5% Nb-doped SrTiO_3_ single crystals as substrate. First, we present data on temperature dependent conductivities and electronic as well as ionic contributions. Second, we describe how different resistive states are formed at high and low temperatures and discuss the underlying mechanisms. Furthermore, we will show that ionic charge carriers play an important role in this context.

## Materials and methods

The slightly Fe-doped SrTiO_3_ (Fe:STO) thin films were deposited by pulsed laser deposition (PLD) from targets with 0.37 mol% Fe (~6.2 × 10^19^ cm^−3^ measured by ICP-MS). The used polycrystalline target was prepared via the mixed oxide route, starting with SrCO_3_, TiO_2_ and Fe_2_O_3_ (Sigma Aldrich, Germany). The powders were homogenized in a mortar and calcinated at 1000 °C for 2 h. Cold isostatic pressing of the powder lead to a green body, which was subsequently sintered at 1200 °C for 5 h in air. During the deposition process the target material was ablated by an excimer laser (λ = 248 nm, Coherent, Germany) with a pulse rate of 5 Hz. Nb-doped SrTiO_3_ single crystals (Nb:STO, 0.5 wt% Nb, 0.5 mm thickness, Crystec, Germany) used as substrate material were heated to 650 °C and held at a constant oxygen partial pressure of 0.15 mbar. By varying the deposition time, layers with thicknesses from about 100 nm to 400 nm were prepared.

The deposited Fe:STO thin films were analyzed by means of X-ray diffraction (XRD), atomic force microscopy (AFM) and transmission electron microscopy (TEM). The layer thickness was measured by digital holographic microscopy (DHM).

**Fig. 1:**
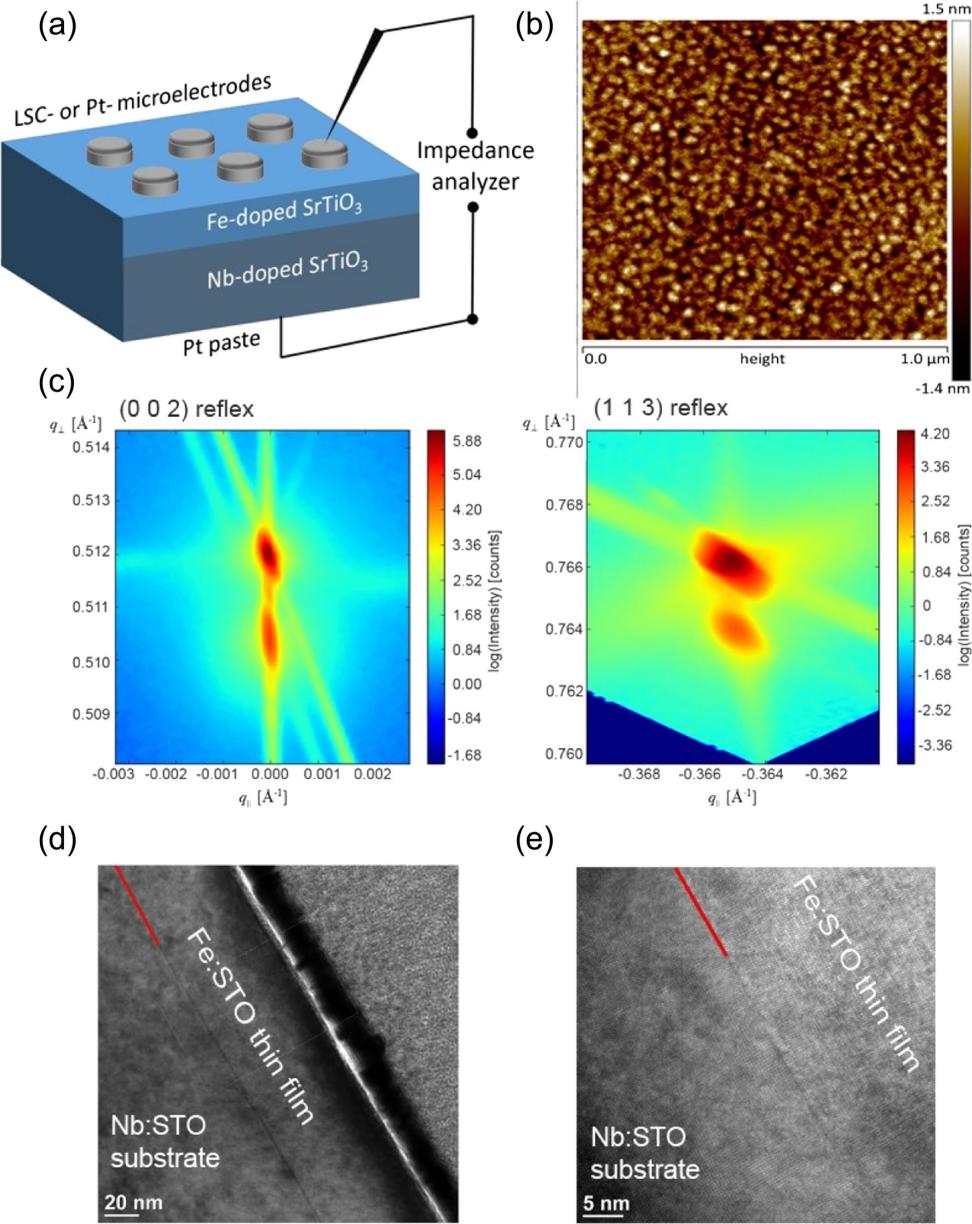
(**a**) Schematic sketch of the investigated sample configuration, with the sandwich-structure of LSC or Pt|Fe:STO|Nb:STO|Pt. (**b**) Atomic force microscopy (AFM) measurements show a smooth surface which is consistent with the results from the other analytical methods. (**c**) Obtained results from high resolution X-ray reciprocal space mapping ([0 0 2] out-of plane reflex, and [1 1 3] in-plane reflex) confirm the results from TEM investigation. (**d, e**) Transmission electron microscopy (TEM) images show a homoepitaxially grown Fe:STO layer on a Nb:STO single crystal. The interface is indicated by the red line, (**e**) is a close-up of the interface region

Electrochemical characterization of the prepared thin films was performed by electrical impedance spectroscopy and *I-V* measurements. All electrical measurements were conducted perpendicular to the surface using the highly conductive Nb:STO substrate as counter electrode. The Fe:STO|Nb:STO interface is at least partially reversible for electrons but completely blocking for oxygen in the investigated parameter regime. (La_0.6_Sr_0.4_)CoO_3-δ_ (LSC) was deposited on top of the Fe:STO layer and served as working electrode. The LSC layer was microstructured by photolithography and subsequently etched with diluted HCl. The resulting circular microelectrodes in the range of 100–300 μm were used with the advantage that multiple measurements (ca. 20) could be performed on one and the same sample. A porous Pt layer was brushed at the bottom of the Nb:STO to achieve a good electrical contact. For all electrical measurements, the microelectrodes were contacted with Pt/Ir-needles. A schematic sketch of the sample configuration is shown in Fig. 1(a). All experiments were performed in ambient air and in a temperature range between 350 °C and 750 °C. Because of the fact that two types of experimental set-ups were used [33], differing in the way the samples were heated, the temperatures are indicated as real sample temperature in case of symmetric heating (T_corr_) or set temperature of the controller in case of asymmetric heating (T_set_). At each temperature the samples were held for a few minutes prior to the measurements, to ensure thermal equilibration. Several temperature cycles were conducted and revealed an excellent reproducibility of the recorded resistivity.

Electrochemical characterization by means of impedance spectroscopy was performed using an Alpha-A High Resolution Dielectric Analyzer connected to a Pot/Gal-Interface (Novocontrol, Germany). The frequency range from 1 MHz to 100 mHz was investigated with a resolution of 10 points per frequency decade. An AC rms amplitude of 20 mV was used for measurements with and without additional bias voltage (up to +/− 500 mV with voltage steps of 100 mV) applied to the LSC working electrode. Because of the fact that LSC is a good ionic conductor the chemical potential of oxygen at the LSC|Fe:STO interface is assumed to be the one of the surrounding atmosphere, regardless of the applied polarity. Therefore, a positive polarity (“anodic”) at the LSC electrode results in a negative polarity (“cathodic”) and thus a lower chemical potential of oxygen at the counter electrode (Nb:STO). The obtained impedance data were parameterized using the complex nonlinear least square (CNLS) fit software ZView3.5 (Scribner, USA).

*I-V* measurements were performed in identical set-ups as for AC measurements using the software WinChem (Novocontrol, Germany). *I-V* curves were recorded in various sweep rates. On the one hand, the measuring time for an *I-V* cycle (+400 mV → -400 mV → +400 mV) was stretched out over hours, to ensure that a steady state condition was reached for each voltage point. In the following, this measurement mode is denoted as “slow” *I-V* curve. On the other hand, *I-V* scans were recorded within seconds, allowing to probe a fixed polarization state (=resistive state). For the fast *I-V* measurements the sample was held at constant bias voltage for at least 5 min (depending on temperature) to reach steady state before the measurement was started. These measurements are denoted as “fast” *I-V* curves.

Isotope exchange experiments were performed at 440 °C in 200 mbar ^18^O_2_ (97.1% isotope enriched, CAMPRO) in a special quartz setup. Subsequent isotope exchange depth profiles were measured on a TOF.SIMS 5 instrument (ION-TOF) using 25 kV Bi_3_^++^ primary ions (ca. 0.03 pA), 2 kV Cs^+^ for sputtering (ca. 90 nA) and a low energy electron gun (20 V) for charge compensation. Details on the used measurement mode (“CBA mode”) are given in Refs [34, 35]. Areas of 70 × 70 μm^2^ were analyzed and sputter crates were 350 × 350 μm wide. Depth information was calculated from sputter currents and sputter time and were additionally referenced by depth measurement using confocal microscopy (Axio CSM 700, Zeiss).

## Results and discussion

### Thin film characterization

A schematic of the sample setup is shown in Fig. 1(a). The surface quality was investigated by AFM in tapping mode as shown in Fig. 1(b). A smooth surface with tiny islands but without any cracks, grain boundaries or larger surface particles was observed for the thin films. The surface roughness was r_a_ ~ 0.2 nm.

Thin films were investigated by high-resolution X-ray reciprocal space mapping (HRXR-RSM). The corresponding reciprocal space map measured close to the (002) diffraction is shown in Fig. 1(c). The substrate peak (higher intensity) is located slightly above the layer peak which emphasizes that the out-of plane lattice parameter for the thin film is slightly greater than the one for the substrate. These findings indicate a slight non-stoichiometry of the Fe:STO thin film and are in agreement with literature [36, 37]. Cation non-stoichiometry in STO can result from preparation at high temperature where a certain equilibrium non-stoichiometry is frozen-in at temperature where cations become immobile. For techniques working at low temperatures such as PLD in this work, A:B cation ratios in perovskites which are not perfectly 1 can occur and lead to a certain low amount of cation vacancies which act as acceptor dopand and increase the lattice parameter.

Transmission electron microscopy (TEM) lamellae were prepared by focused ion beam (FIB). TEM cross section images with two different magnifications of the entire 100 nm Fe:STO layer (Fig. 1(d)) and a close-up view of the Nb:STO|Fe:STO interface (Fig. 1(e)) are shown. From the TEM images, unobstructed homoepitaxial growth is clearly shown.

### Electrical conductivity of the thin films

**Fig. 2:**
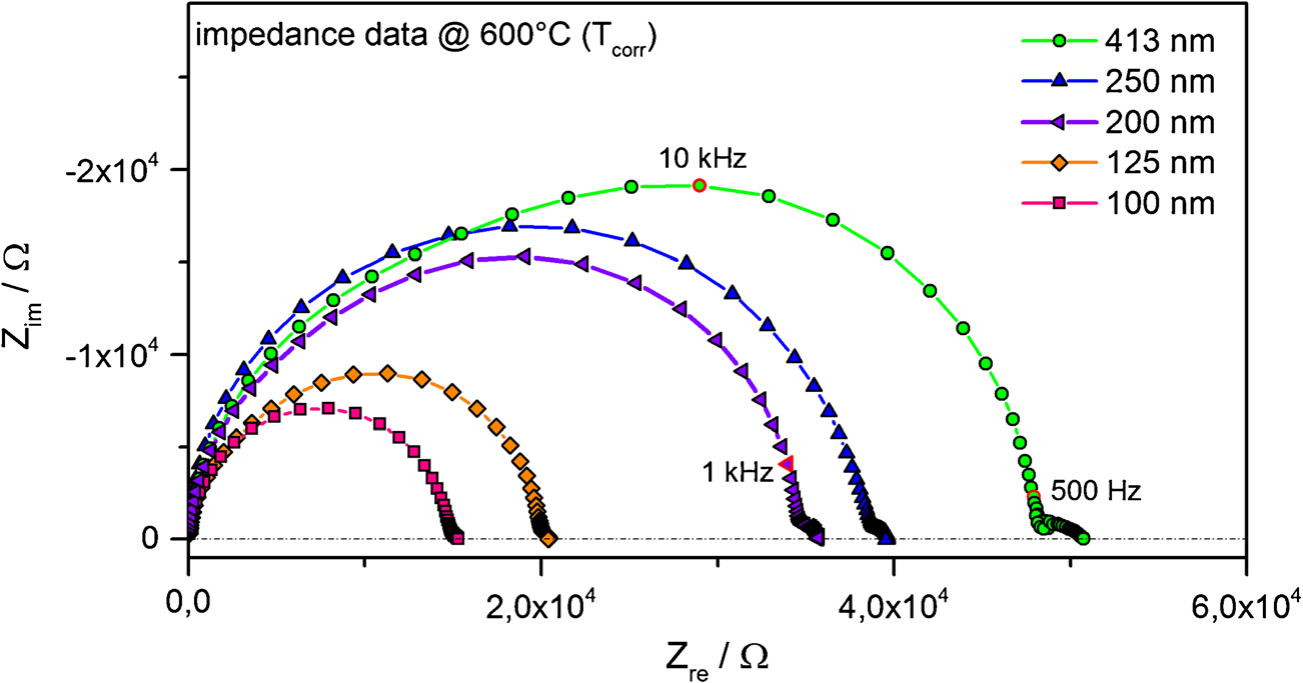
Impedance spectra for 5 different Fe:STO layers in the range between 100 and 413 nm. Each layer was deposited on Nb:STO single crystals. LSC on top of the thin films served as micro-structured electrode material. Impedance spectroscopy measurements were conducted at 600 °C (T_corr_) on microelectrodes with 300 μm in diameter

**Fig. 3:**
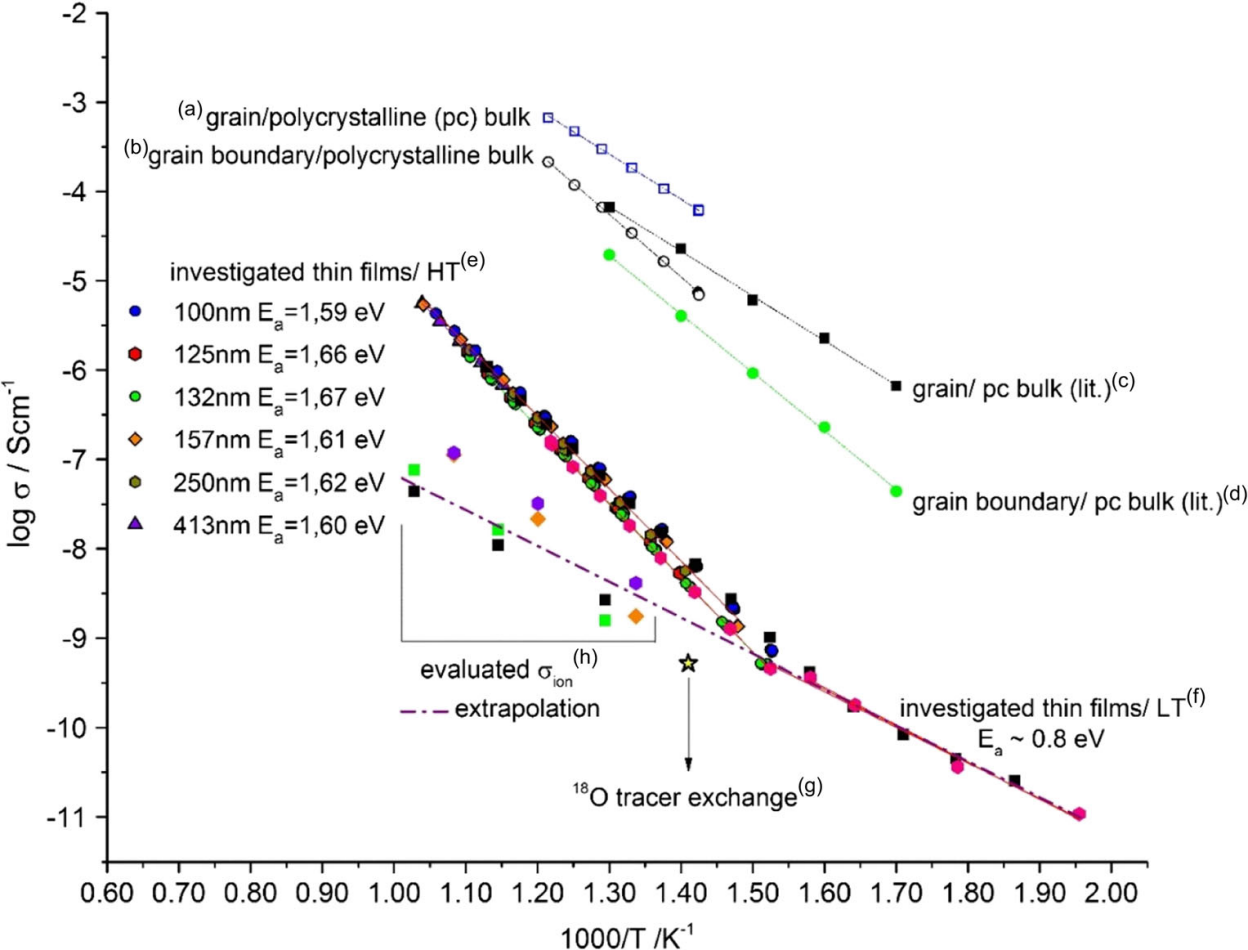
(**a**) and (**b**) are conductivity values for bulk and grain boundary of the polycrystalline target material used in this study, (**c**) and (**d**) are literature values for bulk and grain boundaries from Ref. [51]. (**e**) Arrhenius behaviour for Fe:STO thin films investigated in this study (high temperature behaviour) with an activation energy of around 1.6 eV, (**f**) low temperature behaviour of the investigated thin films with a changed activation energy of around 0.8 eV. (**g**) Conductivity value evaluated from the tracer diffusion coefficient obtained by ^18^O tracer experiments and subsequent SIMS analysis. (**h**) Values for the ionic conductivity, which was gained by evaluating the appearing loop or second semicircle from bias measurements

The thin films were investigated by impedance spectroscopy using (LSC) microelectrodes as top contacts according to the schematic in Fig. 1(a). Nyquist-plots of the impedance response of Fe:STO thin films of different thickness recorded at 600 °C are shown in Fig. 2. Two features are clearly visible: A large, partly distorted semicircle at intermediate frequencies and one small shoulder at low frequencies. A clear dependence of the resistance of the large semicircle on film thickness can be seen. Indeed, a direct scaling of the resistance with Fe:STO film thickness was observed in the investigated temperature range. This is also visible from Fig. 3 (graphs marked “e” and “f”), where the conductivity at a certain temperature is independent of the film thickness. This is a strong indication that the large semicircle is the resistive response of the entire Fe:STO layer, despite some distortions of the arc. Additional evidence comes from the capacitance calculated for undistorted large semicircles, which agrees well with the geometrical capacitance of the Fe:STO thin film expected for a permittivity ε = 160 at 300 °C [38]. The small low frequency feature (in the 10–100 Hz range for the 413 nm layer, Fig. 2) has a larger capacitance in the order of μF/cm^2^. No evidence of significant resistive contributions of the p-n junction at the Fe:STO|Nb:STO interface or any space charge region at the LSC electrode were found; such resistive contributions would not scale with the thickness of the thin film. A metal-insulator transition in a thin zone at the surface of Nb:STO [39] could be a possible cause of the small low frequency arc, but can be excluded as dominating resistive contribution due to its observed scaling with thickness.

Upon varying the temperature, the Fe:STO impedance response remains one single arc in the investigated temperature range 350 °C–750 °C. However, plotting this resistance in an Arrhenius diagram (Fig. 3) a clear transition at about 390 °C is visible. At higher temperatures (“e”) the investigated Fe:STO thin films show an activation energy of the conductivity of about 1.6 eV, while at lower temperatures (f) the activation energy is only about 0.8 eV. For comparison, also a polycrystalline Fe:STO sample (i.e. a pellet used as PLD target) was investigated by impedance spectroscopy. Two semicircles were found and in agreement with literature interpreted as grain and grain boundary resistances. From a brick layer model analysis [40] we obtain the grain and grain boundary conductivities shown in Fig. 3(a), (b). The values are in acceptable agreement with literature data for polycrystals with a similar doping level see Fig. 3(c), (d) [41].

It is clearly visible that the thin films investigated in this study show much lower conductivity values than macroscopic polycrystalline samples [41–43]. Also the activation energies do not agree. Two possible causes for the observed difference between epitaxial thin films and single crystals are: (i) strain effects from thin film growth or (ii) a high dislocation density in the thin films so that the resulting space charges in the vicinity of each dislocation overlap and affect the whole film. It is beyond the scope of this paper to further clarify this point (e.g. by isotope tracer experiments over the whole temperature regime, but we assume that the high and low temperature conductivities of the thin films can be attributed to electronic (high T) and ionic (low T) conduction. Arguments in favor of the first point (electronic at high T) are earlier measurements on the bias dependence of the spectra and *I-V* curves [29]. There, a model based on electron conduction and redistribution of ions upon bias was able to consistently explain very unusual impedance spectra. Some aspects of this model are described below and used to further confirm consistency of our interpretation in terms of electron or ion conduction. A second argument fitting to the interpretation of electrons, as the conducting species at higher temperatures is the high activation energy (ca. 1.6 eV) which is about half the band gap. This, however, would suggest that the thin films are electronically close to the intrinsic state. The interpretation of the low temperature conductivity as ionic is supported by the activation energy, which fits to the migration barrier assumed for SrTiO_3_ [41]. Two further arguments result from the additional measurements described below.

### Ionic conductivity of the thin films from isotope exchange depth profile

Ionic conductivity in strontium titanate is possible via oxygen vacancies, represented by *δ* in the stoichiometry SrTiO_3-δ_. These can be created or filled via the oxygen exchange reaction.

1 $$ {O}_O^{\times}\leftrightarrow {V}_O^{\cdotp \cdotp }+2{e}^{\prime }+\frac{1}{2}{O}_2 $$

Furthermore, the concentration of oxygen vacancies can be increased by acceptor doping.

2 $$ {Fe}_2{O}_3\overset{{ Ti O}_2}{\to }2{Fe}_{Ti}^{\hbox{'}}+3{O}_O^x+{V}_O^{\cdotp \cdotp } $$

**Fig. 4:**
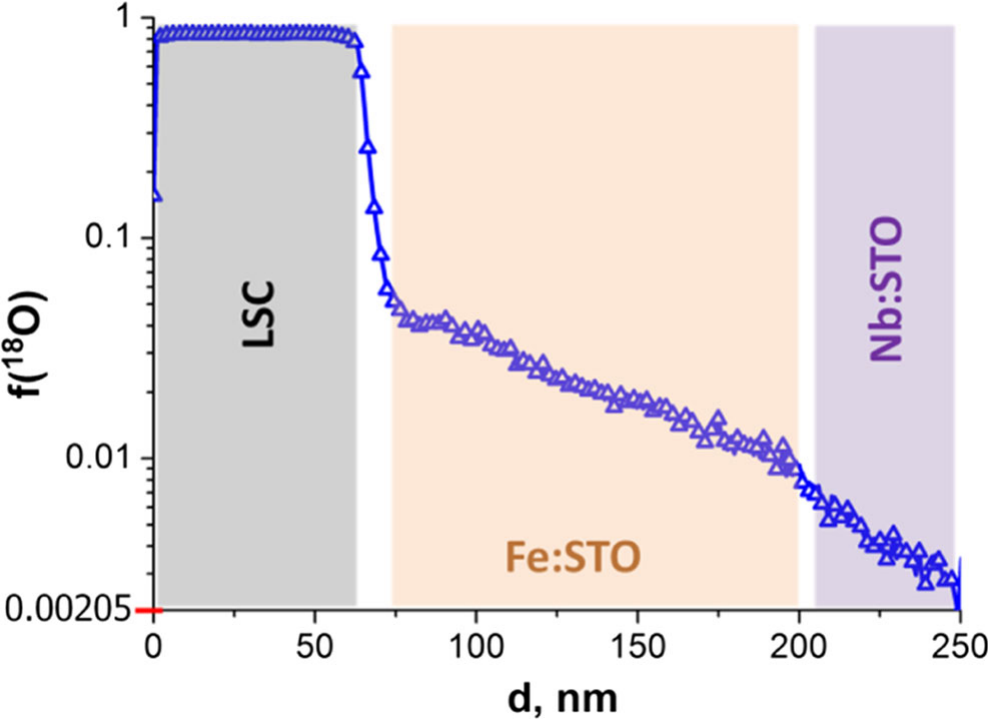
A typical isotope exchange depth profile of LSC/Fe:STO/Nb:STO sample which was annealed in 200 mbar ^18^O_2_ at 440 °C for 1080 min. The diffusion coefficient *D* ^*^ of Fe:STO was calculated from the profile by finite element calculations

Ionic conductivities can be calculated from oxygen tracer diffusion coefficients measured via isotope exchange depth profiling using time-of-flight secondary ion mass spectrometry (ToF-SIMS). Fig. 4 displays an isotope exchange depth profile (IEDP) found after thermal isotope exchange at 440 °C for 1080 min. The profile reveals that a LSC|Fe:STO interfacial diffusion barrier leads to an isotope concentration step between LSC and Fe:STO. However, the nature of this highly resistive interface is not clear. Analysis of the entire profile requires numerical calculation (finite elements method) to obtain the tracer surface exchange coefficient k* and the tracer diffusion coefficient D* in LSC and SrTiO_3_ simultaneously in one fit. In these numerical calculations, also a thin zone at the LSC|Fe:STO interface with reduced diffusivity and a finite diffusion coefficient in Nb:STO was used. The reason for the latter is still under debate. For the comparison of tracer diffusion coefficient *D* ^*^ and bulk electrical conductivity due to oxygen diffusion, it is necessary to calculate the electrical diffusion coefficient *D* ^*q*^. *D* ^*q*^ relates to *D* ^*^ via the Haven ratio according to Eq. 3.

3 $$ {D}^q\cdot H={D}^{\ast } H\approx {f}_s=0.69 $$

The Haven ratio can be assumed to be similar to the correlation factor f_s_ which is 0.69 for perovskites, which was used for this study [44, 45]. Via the Nernst-Einstein equation

4 $$ \sigma =\frac{D^q{c}_O{z}^2{c}^2}{kT} $$

it is then possible to convert the diffusion coefficient into ionic conductivity values. The resulting value is plotted as star shaped data point in Fig. 3 and is close to the extrapolation of the low temperature (ionic) conductivity and thereby support the given interpretation.

### DC-voltage in the high temperature regime – Migration of oxygen vacancies enforces a redistribution of electronic charge carriers

**Fig. 5:**
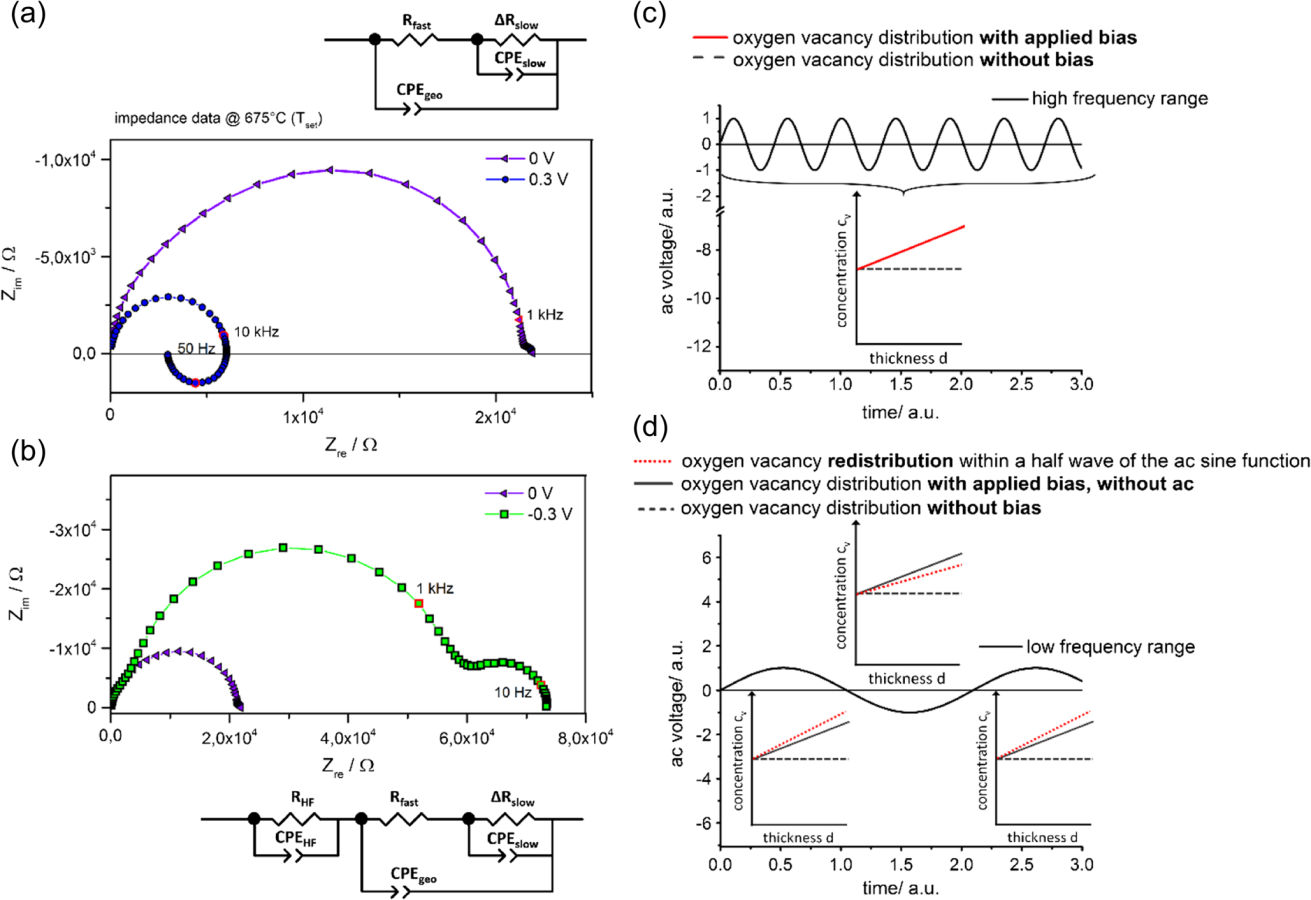
(**a**) Anodic bias voltage results in a smaller high frequency resistance compared to the unbiased measurement. Furthermore a second feature appears in the low frequency range. For the anodic case this manifests itself in an “inductive” loop, here measured at 675 °C (T_set_). (**b**) Impedance spectra for the cathodic case often show an increased high frequency resistance with the additional appearance of a low frequency second semicircle. For both cases the equivalent circuits used to fit the data are shown. A schematic sketch for the high frequency as well as for the low frequency situation are shown in (**c**) and (**d**). The red curves indicate the oxygen vacancy distributions probed by the impedance measurements depending on the applied frequency and time

As described before, oxygen vacancy formation is driven by the exchange of oxygen as well as by acceptor doping. The corresponding set of defect chemical equations for acceptor doped bulk SrTiO_3_ are well established in literature [41]. Therefore, it is possible to calculate the electronic and ionic charge carrier mobilities and concentrations for a defined situation (oxygen partial pressure, temperature and doping level). The investigated parameter regime of ambient air, a temperature range from 350 to 750 °C and a doping concentration of 0.37 mol% results in a defect chemical situation where oxygen vacancies are the majority charge carriers in the bulk [7]. For our films we expect in accordance that oxygen vacancy concentrations are larger than electronic charge carrier concentrations in the whole temperature range. However, they determine the overall conductivity only at low temperatures, where only very few electronic charge carriers exist, while the high temperature regime reflects electronic conduction due to the much larger mobilities compared to ions. In such a situation, a novel electrochemical method for determining the ionic conductivities can be applied. This method was introduced in Ref. [29] and is based on potentiodynamic electrical impedance spectroscopy, which means that different DC biases are applied during the AC measurement. Both bias directions lead to non-trivial impedance spectra as indicated in Fig. 5.

A positive bias applied to the top electrode (LSC) leads to a feature at low frequencies (Fig. 5(a)) which resembles an “inductive” loop sometimes found as measurement artifact. However, this additional feature is not an artifact and can be parameterized using a negative resistance *ΔR*_*slow*_ and a negative capacitance *C*_*slow*_, here represented by a constant phase element *CPE*_*slow*_. A negative bias often leads to a semicircle-like feature at low frequencies (Fig. 5(b)). As can be seen from Fig. 2 as well as Fig. 5(a), (b), the low frequency features do not occur in impedance measurements without bias. The equivalent circuits which were used for fitting the bias dependent impedance data are shown in Fig. 5(a), (b). The additional *R*_*HF*_ *CPE*_*HF*_ element used for negative bias takes account of the more pronounced high frequency shoulder found in such cases. A detailed derivation of these circuits and of the defect chemical reasons behind the different bias features is given in Ref [29]. The features appearing under bias are caused by the different response times of electronic and ionic charge carriers adapting to the bias non-equilibrium. At high frequencies, the current response from electrons or holes is probed for a given distribution of ionic defects. Ionic charge carriers are not able to move substantial distances within a single half wave of the AC sine function. At low frequencies, long range movement of oxygen vacancies takes places also within a single AC half wave. That means, at low frequencies the redistribution of oxygen vacancies over the entire film thickness leads to a change in the local stoichiometry and resistivity of the material. This is sketched in Fig. 5(c), (d). These changes of the electronic currents cause the additional loops or semicircles at low frequencies. Hence, the appearance of the low frequency feature in impedance measurements can give information on the ionic resistance, although no ionic contribution to the current is directly probed. Following the model in Ref. [29], this ionic resistance R_ion_ determines the low frequency capacitance according to:

5 $$ {C}_{slow}={C}_{el}\frac{R_{ion}}{\Delta {R}_{slow}}. $$

*ΔR*_*slow*_ and *C*_*slow*_ are fit parameters describing the additional low frequency loop or arc and were evaluated for the entire range of bias voltages. When assuming an ion blocking interfacial capacitance *C*_*el*_, the ionic resistance becomes accessible via Eq. 5. The peak frequency of the low frequency loop or arc depends on R_ion_ and C_el_ according to:

6 $$ \omega =2\pi f=\frac{1}{R_{ion}{C}_{el}}. $$

**Fig. 6:**
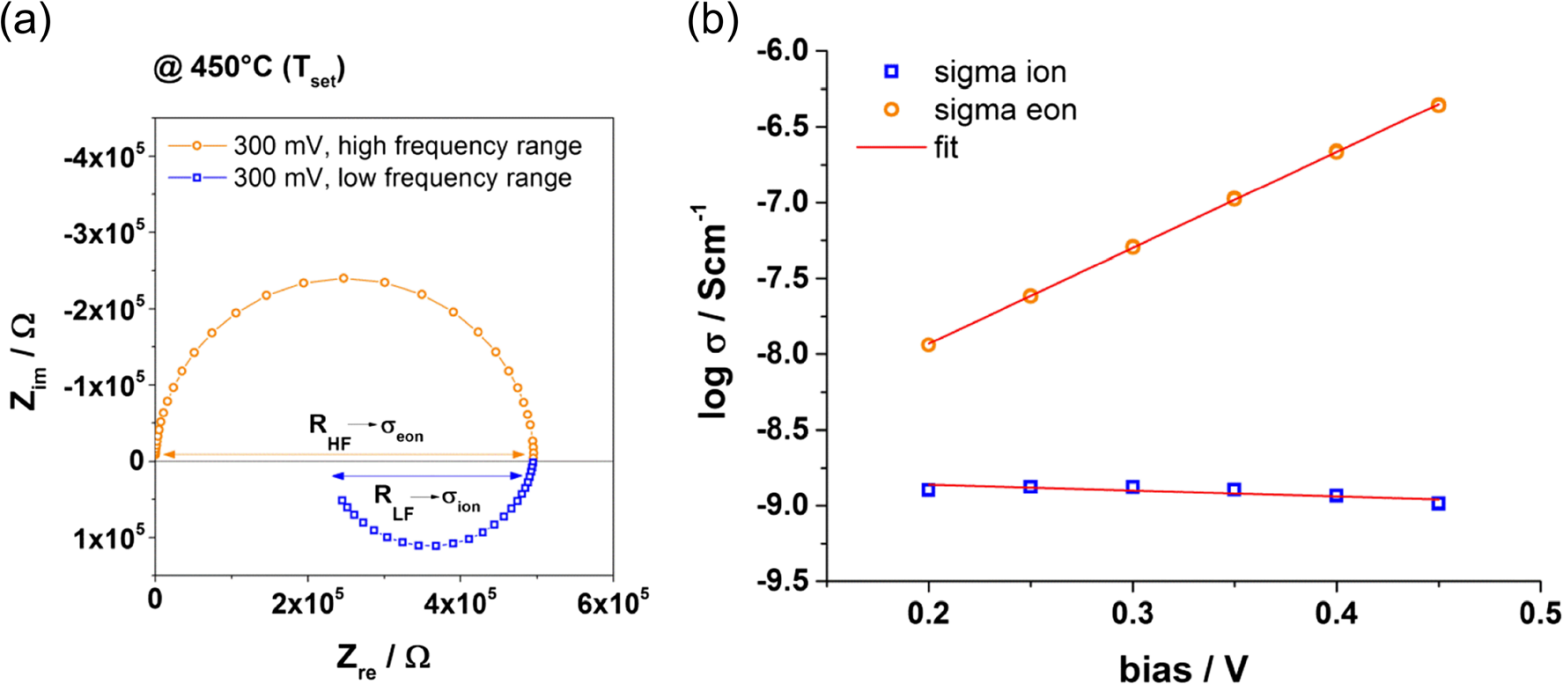
(**a**) Impedance measurements under bias give an additional low frequency feature containing information on the electronic conductivity in the high frequency range and on the ionic conductivity in the low frequency range. (**b**) Using Eq. 6 allows the calculation of the ionic conductivity for varying bias voltages. The obtained values are plotted in comparison to the electronic conductivity. For the ionic fraction no distinct dependence on bias can be found. Extrapolation of the data to 0 V gives the ionic conductivity for the non-biased case

As it can be seen from Fig. 6 the deduced ionic conductivity has only a minor dependence on the bias. This is in good agreement with the assumption that oxygen vacancies are the majority charge carriers. In other words, the redistribution of ionic charge carriers as a reaction to the applied bias, is small in comparison to the total concentration of oxygen vacancies within the thin film. The distinct response of electronic charge carriers on the bias and thus on the ionic redistribution can be explained by the small concentration of these charge carriers. To get information on the non-biased ionic conductivity, the ionic conductivity values in Fig. 6 are extrapolated to 0 V resulting in an ionic conductivity of 1.7 × 10^−9^ S/cm for 450 °C. When applying this procedure to the different film thicknesses at varying temperature, we obtain the ionic conductivities shown in Fig. 3(h). An ion blocking interface capacitance of ~20 μF/cm^2^ was assumed according to a relative permittivity of ε ~ 100 and a typical ionic space charge thickness of ~4 nm. Despite some scatter, the data are in excellent agreement with the extrapolation of the low temperature zero bias conductivities. This agreement further supports the interpretation of low temperature conductivities as ionic conductivities. Electronic conductivities can still be evaluated from the high temperature arc and are strongly bias dependent in agreement with our model.

At elevated temperatures only a constant applied bias voltage can sustain the redistributed state of oxygen vacancies. Changing or removing the voltage immediately leads to an adaption of the charge carrier distribution to the new situation. This makes probing of a certain resistive state difficult. “Slow” *I-V* curves conducted at these temperatures probe a different vacancy distribution and thus a different resistive state in each voltage point. The continuous change of the voltage during such DC measurement results in a continuous charge carrier redistribution within the layer. In addition to the electrically driven movement of the ionic charge carrier, their increased mobility due to thermal activation is responsible for the fast changes of the charge carrier distribution. At high temperature, there is a maximum time allowed for probing a given resistive state by a “fast” *I-V* curve without changing the state. This time was estimated from the impedance spectra: a frequency higher than the onset of the low frequency feature (loop or arc) has to be taken to avoid redistribution of ions. Thus times <ms result above 700 °C. Consequently, in the higher temperature regime we could not access particular resistive states via *I-V* measurements.

### DC voltage in the intermediate temperature regime - addressing resistive states of Fe:SrTiO_3_ by affecting the defect chemistry

*I-V* curves were also measured for the intermediate temperature regime (<500 °C), see Fig.3. For “slow” *I-V* curves the scan rate corresponds to a frequency smaller than the frequency points for which in impedance spectra the real axis is reached for low frequencies. Only then, a new steady state can be ensured in each voltage point. The most important defect species to form the new steady state are oxygen vacancies, however, for a more complete picture complex defect associates and negative frozen-in metal vacancies need to be considered [30, 46]. By taking into account that the mobility of all ionic charge carriers is reduced at lower temperatures, it becomes obvious that the required time for “slow” *I-V* curves increases with decreasing temperature and may reach the range of days. On the other hand, the reduced mobility of oxygen vacancies now offers the opportunity to probe the charge carrier distribution in a “frozen-in” situation and thus particular resistive states. Such states can be realized by applying a bias voltage for a finite time. Within this time a constant current is reached which is associated to a distinct charge carrier distribution. Other than at high temperature, this embossed state can now be probed by “fast” *I-V* measurements: The appropriate time for “fast” *I-V* curves is again evaluated from impedance spectra conducted at a given bias voltage, see above. At lower temperatures, the required sweep rate shifts into the range of seconds and is therefore experimentally accessible.

**Fig. 7:**
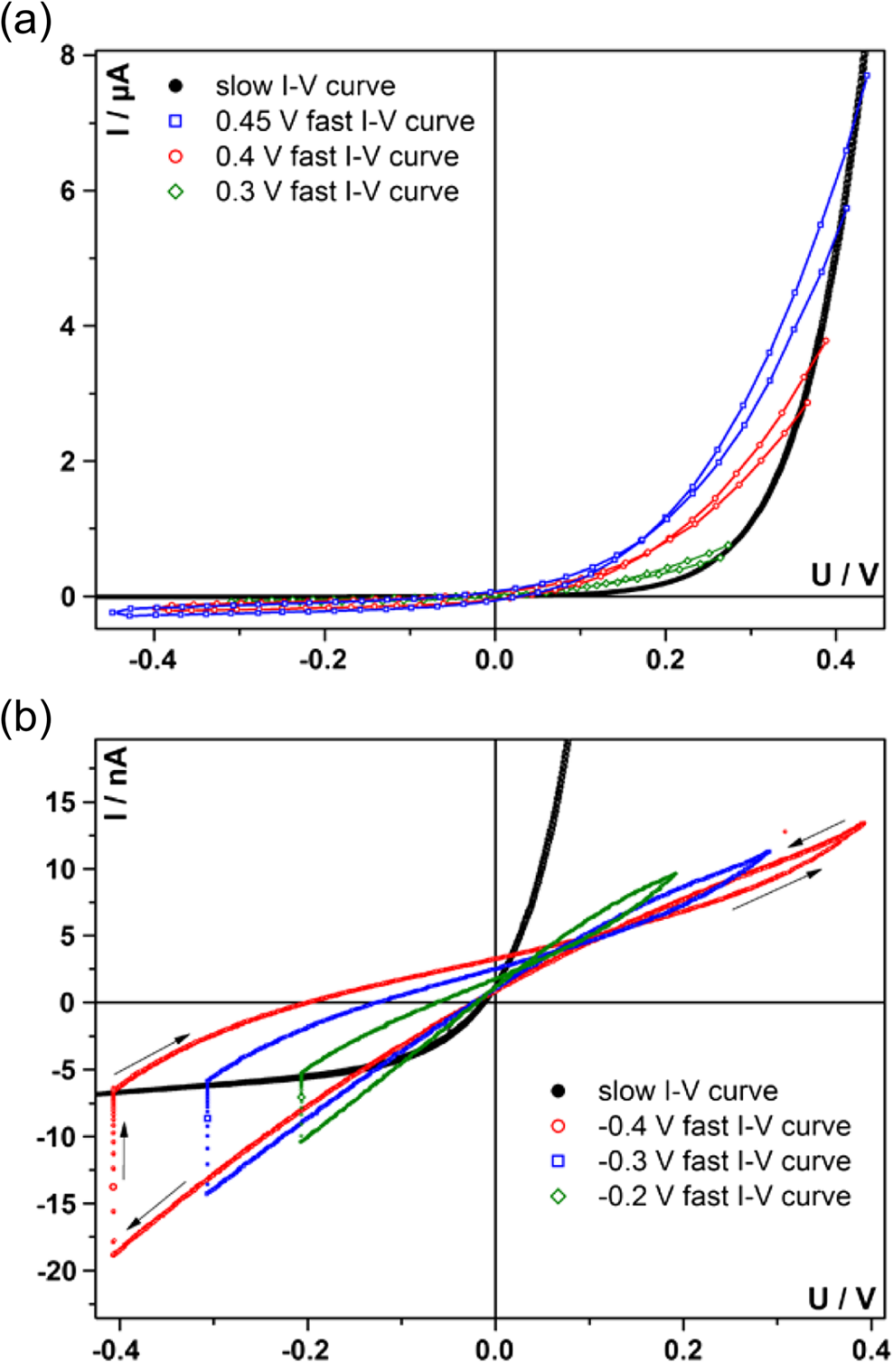
(**a**) Fast *I-V* curves (sweep rate 25 V/s) measured at 425 °C, in the anodic regime, are compared with the slow *I-V* curve (0.05 mV/s). Depending on the applied voltage, different resistive states are probed. The same behaviour is observed for the cathodic regime (**b**). In particular the fast *I-V* curves (sweep rate 1 V/s) starting in the cathodic regime are still affected by the bias induced ion motion. This is reflected by some hysteresis, which cannot be completely prevented

Such fast conducted *I-V* measurements reflect the properties of dedicated resistive states. Different resistive states were adjusted by different voltages and probed for a 200 nm sample at 425 °C (T_set_), which is shown for the anodic case in Fig. 7(a) and for the cathodic case in Fig. 7(b). For comparison, the slow *I-V* curve for the same temperature is also given. The slight voltage off-set for the open circuit condition (slow *I-V* curve) can be attributed to the asymmetric heating /thermovoltage within the experimental set-up. The off-sets of the fast *I-V* curves are predominantly due to hysteretic effects.

It becomes obvious from Fig. 7 that the established resistive state can be probed by fast *I-V* curves but does not sustain more than one cycle, which is in contrast to non-volatile resistive switching at room temperature. Already conducting a cycle after an anodic bias, i.e. sweeping from positive to negative and back to positive voltage results in a small change of the measured current. In other words, the charge carriers still slightly adapt to the changing voltage, which results in a small redistribution accompanied with a changed current. Subsequently applied constant voltage and repeating the measurement after reestablishing steady state yields again the same *I-V* curves. Irreversible effects could therefore be excluded as cause for the observed resistance changes. This behavior was found despite the fact that the appropriate sweep rate for a “fast” *I-V* curve is easily accessible in the anodic case, due to the fact that impedance data points cross the real axis. Therefore, the frequency point without phase shift can be exactly determined and transferred into a sweep rate. The slight change in the current probably originates from the fact that this appropriate sweep rate depends on bias voltage which changes during the measurement.

**Fig. 8:**
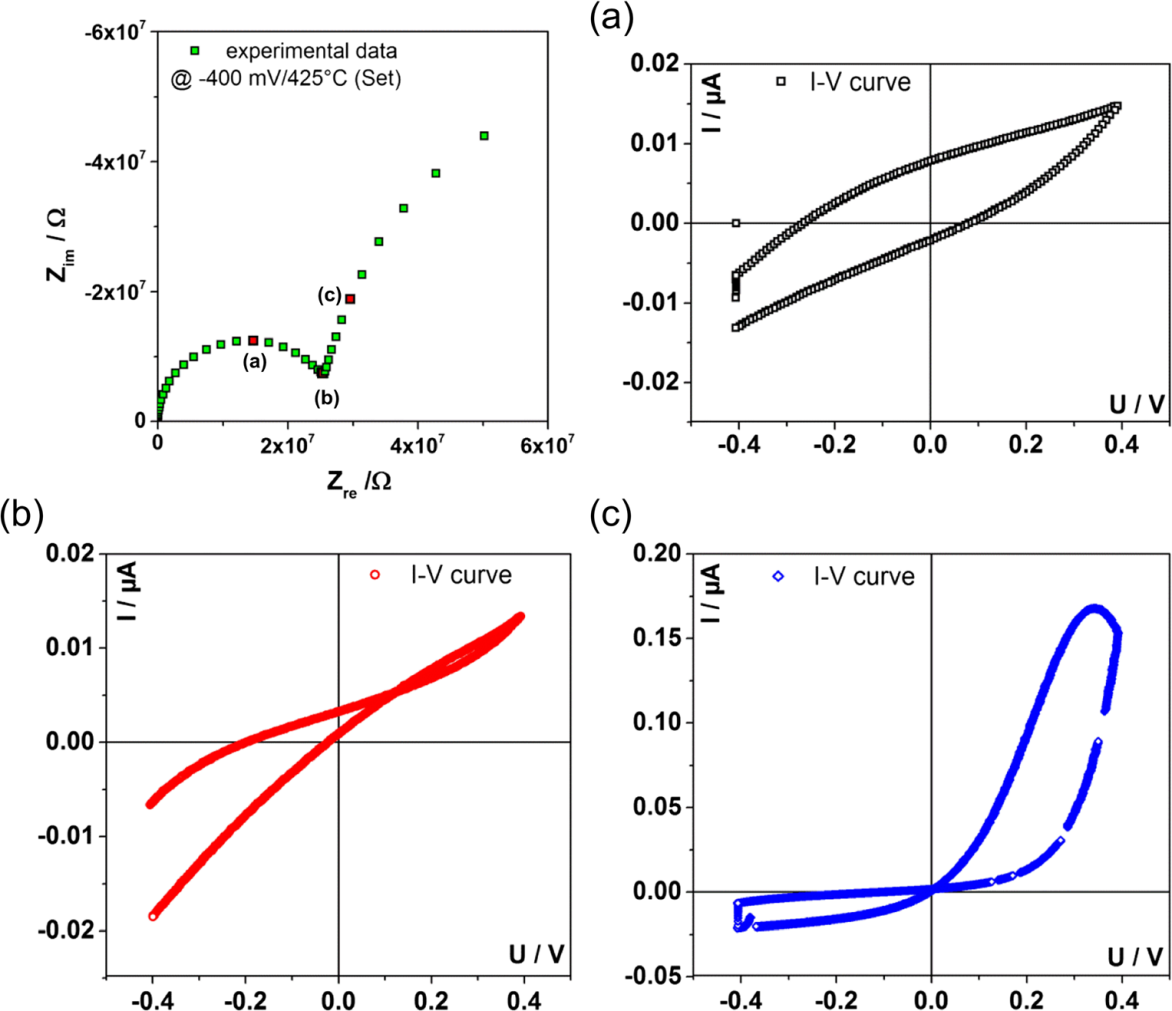
An impedance spectrum recorded at 424 °C (T_set_) and −400 mV bias voltage is shown. Three frequency points are indicated by (**a**), (**b**) and (**c**). Corresponding to each frequency point “fast” *I-V* curve were measured. The sweep rate in point (**a**) was too high, which leads to an open hysteresis curve. With the corresponding sweep rate from point (**b**) a comparatively undisturbed “frozen-in” state of the defect chemistry can be probed. Shifting the sweep rate to point (**c**) gives an *I-V* curve that additionally probes the redistribution of ionic charge carriers

We could further monitor that fast conducted *I-V* curves starting in the cathodic regime strongly depend on the sweep rate. This can be attributed to the fact that there is no intercept with the real axis in the corresponding impedance spectrum, see Fig. 8. The two recorded semicircles overlap, which makes it hard to evaluate the appropriate frequency, as there is no frequency without phase shift in that range. Therefore a sweep rate without additional effects does not exist. Also for the most appropriate frequency, the recorded fast *I-V* curve in Fig. 8(b) does not reflect a true frozen-in defect chemical situation. It rather contains contributions either of the charging of the geometrical capacitance, more pronounced for higher sweep rate, see Fig. 8(a), or the redistributing of oxygen vacancies due to the AC signal for lower sweep rates shown in Fig. 8(c).

Regardless of the observed differences between the anodic and cathodic regime, bias induced motion of oxygen vacancies plays an important role in the establishment of resistive states at these temperatures.

### DC voltage at room temperature - resistive switching

In most oxygen ionic devices, temperatures above 300 °C or higher are typically necessary for devices to function. The reason for that is the relatively high migration barrier that oxygen ions have to overcome in most materials. All alterations of the resistive states at high temperatures are enabled by the substantial mobility of ionic charge carriers. At room temperature it is also possible to alter resistive states by redistributing ions, however, a large driving force in the form of a strong electric field is necessary to overcome the very low ionic mobilities. Via such an effect of large electric fields, non-volatile resistive switching of SrTiO_3_ is possible at room temperature in ambient conditions [23, 47]. For surpassing today’s transistor-based memory technology, this requires long retention of more than 10 years of the resistive states at low bias and fast switching within nanoseconds at large electric bias. This “voltage-time dilemma” [23] is one of the central challenges for using resistive switching as novel memory technology.

**Fig. 9:**
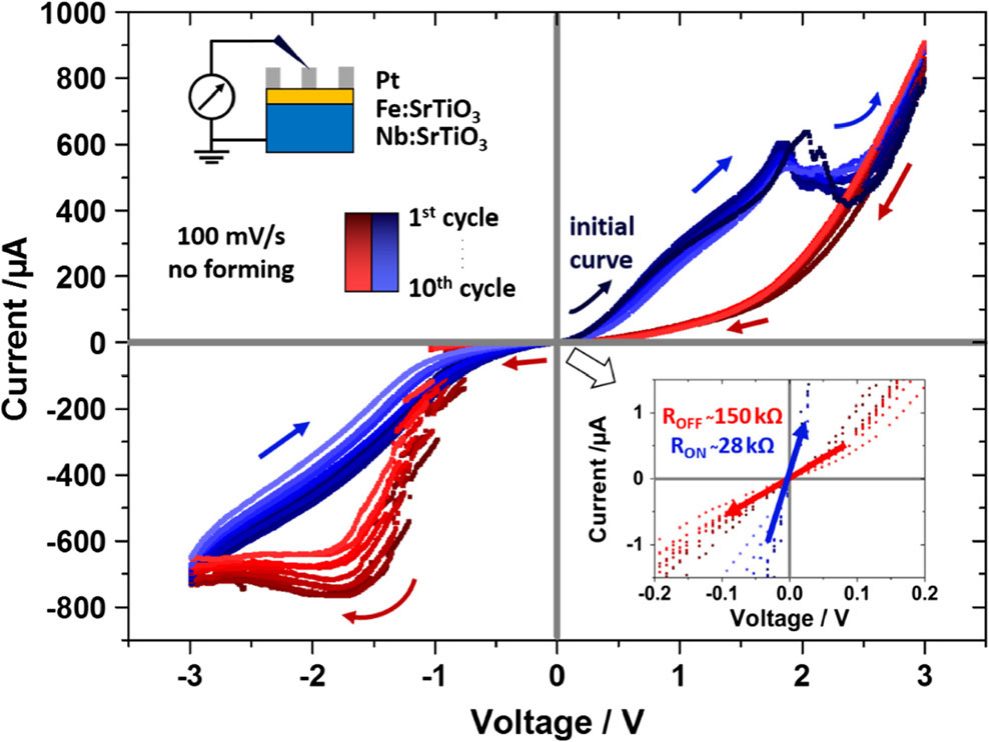
Current–voltage curves of Pt|Fe:STO|Nb:STO structures with Pt micro-electrodes measured at ambient air and room temperature. Ten consecutive cycles with typical hysteretic curves are shown. The inset gives a magnification close to the origin, which was used to determine R_OFF_ and R_ON_

Resistive switching was already shown in literature for SrTiO_3_-based devices [25, 26, 48–50]. Therefore, the resistive response of our Fe:SrTiO_3_ thin films was also investigated under ambient conditions at room temperature and higher electric field strengths using differently sized microelectrodes of Pt and LSC. In Fig. 9, ten current voltage cycles (±3 V, 100 mV/s) of a 200 nm Fe:STO thin film with 200 μm diameter Pt top electrode are exemplarily shown. Resistive switching is reproducibly observed over all 10 cycles, however, a slight drift of the currents in the ON- and OFF-state to lower values is found over the 10 cycles. The average resistance values of the two resistive states at low voltages were *R*_*ON*_ = 28 kΩ, *R*_*OFF*_ = 150 kΩ and the R_OFF_/R_ON_ ratio is consequently about 5.

**Fig. 10:**
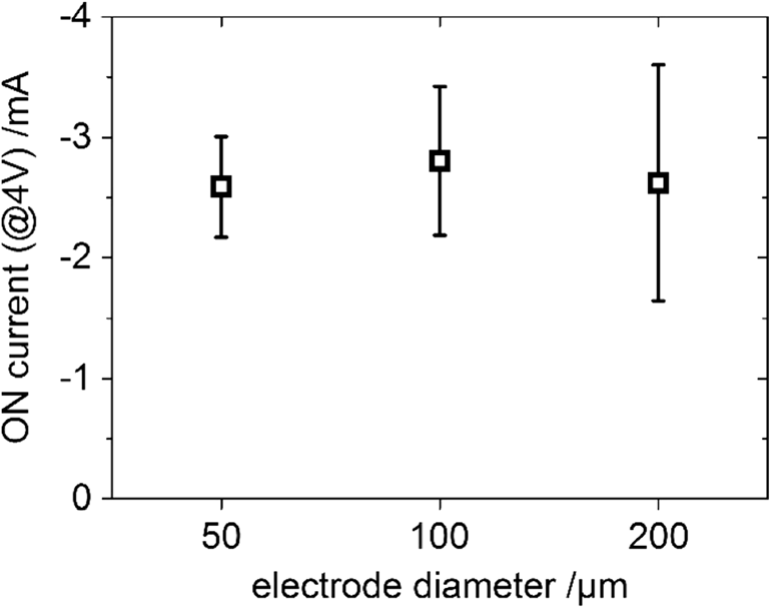
ON currents of resistive switches with Pt-top electrodes measured at −4 V applied bias. No dependence of the current on the electrode diameter is observed, which is expected if the whole current flows through a single conductive filament of similar size

In a following screening study, different circular Pt or LSC microelectrodes with 50–200 μm diameter were investigated. A large number of microelectrodes (> 60) was measured, each by 3 cyclic voltammetry cycles of ±4 V with 200 mV/s sweep rate, starting with positive bias applied to the top electrode. All investigations were done without pretreatment or a forming step as often described in literature. Several results were obtained from this screening study: (i) No hysteretic resistive switching curves were observable with LSC as top electrode. (ii) 16 out of 40 (40%) Pt top electrodes showed reproducible resistive switching curves in all cycles (cf. Fig. 9), while the remaining film regions with Pt top electrodes typically remained in a high resistive state. (iii) No dependence of the low resistive (ON) state on the electrode diameter was found. ON currents were about −2 to −3 mA at −4 V bias for the different electrode sizes as shown in Fig. 10 for all electrodes exhibiting resistive switching. (iv) A higher fraction of samples showing resistive switching was found for larger top electrode diameters (3/10, 30% for 50 μm; 6/15, 40% for 100 μm, 7/15, 47% for 200 μm).

(i) The reasons why resistive switching does not occur in the LSC|Fe:STO|Nb:STO devices is not immediately clear. The fact that the current-voltage curves with LSC are similar to those in the ON-state with Pt-electrodes leads to the conclusion that switching from ON to OFF state is not possible with LSC. Two main differences exist between Pt and LSC top electrodes: the band energies and therefore the resulting space charge in Fe:STO close to the interface and the fact that LSC is a better oxygen ion conductor than Pt. Either of those properties seems to prevent interrupting the low resistive ON state to form the OFF state at large positive biases with LSC.

(ii),(iv) The typical resistive switching mechanism in SrTiO_3_ is via conductive filaments as described in literature. Characteristic features of this mechanism such as current fluctuations at switching from ON to OFF (+1.5 V to +2.5 V in Fig. 9) and from OFF to ON (−0.9 V to −1.5 V in Fig. 9) are also directly observable from our current-voltage curves. The fact that the ON state is independent of the electrode size as shown in Fig. 10 is another strong indication that switching is filament-type: Independent of the electrode size, just one conductive filament enables a current of about 2–3 mA at 4 V in the ON state.

(iii) The rather low total success rate for resistive switching in Pt|Fe:STO|Nb:STO and the dependence of this rate on the electrode size are connected. Resistive switching without a forming step typically requires some critical defects or proto-filaments which can form a conductive filament upon switching. In the measured samples we expect very few of such critical defects due to the homoepitaxial growth. Indeed, more often such critical defects seem to be present in SrTiO_3_ regions beneath electrodes with larger size, which explains their higher rate of resistive switching. The majority (60%) of investigated microelectrodes remained in a high resistive state and didn’t show switching. We interpret those film regions to be free of such critical defects. This, however, does not mean that they cannot be utilized for resistive switching. Forming procedures (high applied currents or voltages over an extended time period) are often used to create proto-filaments in thin films that would otherwise not show resistive switching. We expect that by such a forming step the number of microelectrodes that show switching would increase significantly.

## Conclusions

Fe:STO thin films of ca. 100–400 nm thickness strongly differ in their conductivity from macroscopic polycrystals of the same composition. A transition from predominantly electronic conductivity with high activation energy (1.6 eV) to ionic conductivity with only 0.8 eV activation is observed in our Fe:STO thin films. This interpretation is also supported by isotope tracer based measurements of the ionic conductivity. At elevated temperatures of 400–700 °C it is shown how defects can be redistributed in SrTiO_3_ by a bias voltage and how different resistive states can be addressed at different timescales. Resulting effects of this redistribution such as inductive loops or extra semicircles in impedance spectra can be comprehensively described and even tiny ionic conductivities in the electronic conducting regime can be successfully calculated via an equivalent circuit model. Moreover, it is shown that resistive switching at room temperature is another mechanism in which the electronic conductivity of SrTiO_3_ is controlled via ionic defect changes. In contrast to higher temperatures, ionic changes do not occur in the whole volume, but in a locally confined conductive filament. The probability of forming such a conductive filament depends on the micro-electrode size, whereas the current flowing in the low resistive state is independent on the electrode size.
